# Recommendation for the definition of postoperative radiotherapy target volume based on a pooled analysis of patterns of failure after radical surgery among patients with thoracic esophageal squamous cell carcinoma

**DOI:** 10.1186/s13014-018-1199-3

**Published:** 2018-12-27

**Authors:** Xiaofei Zhang, Xi Yang, Jianjiao Ni, Yida Li, Liqing Zou, Li Chu, Xiao Chu, Fan Xia, Zhengfei Zhu

**Affiliations:** Department of Radiation Oncology, Department of Oncology, Fudan University Shanghai Cancer Center, Shanghai Medical College, Fudan University, 270 Dongan Road, Xuhui, Shanghai, 200032 China

**Keywords:** Thoracic esophageal squamous cell carcinoma, Postoperative radiotherapy

## Abstract

**Background:**

Elective use of radiation therapy to treat regionally involved lymph nodes (LNs) after radical surgery for esophageal squamous cell carcinoma (ESCC) is controversial. We studied metastasis patterns through a pooled analysis of published results to guide post-operative radiotherapy (PORT) target designation.

**Methods:**

We searched the MEDLINE database for literature published from May 1977 to March 2018, and found 14 relevant original studies that included 2738 patients with thoracic ESCC. We calculated probabilities of recurrence and metastasis in local (including anastomoses and tumor bed), LNs and distal areas.

**Results:**

Recurrence rates were 1.88% for local, 13.18% for distal, and 22.16% for LNs. Within LNs, recurrence rates were cervical/supraclavicular: 37.69%, upper mediastinal: 44.30%, middle mediastinal: 21.81%, lower mediastinal: 2.57%, abdominal paraaortic: 25% and upper abdominal: 9.56%. Whereas cervical/supraclavicular and upper mediastinal LNs had the highest recurrence rates, abdominal LNs also had high recurrence rates in patients with lower thoracic ESCC.

**Conclusions:**

PORT volume should include the cervical/supraclavicular and upper mediastinal LNs for all thoracic ESCC, and abdominal paraaortic LNs for lower thoracic ESCC. Anastomoses and tumor beds should not be included in the PORT volume if they are not adjacent to the PORT-LN regions. Upper abdominal LNs might not necessarily be included in the PORT volume for thoracic ESCC.

**Electronic supplementary material:**

The online version of this article (10.1186/s13014-018-1199-3) contains supplementary material, which is available to authorized users.

## Introduction

Although incidence rates for esophageal adenocarcinoma have been increasing in several Western countries, esophageal squamous cell carcinoma (ESCC) is the most common histological type in Asian countries, such as China, where it accounts for more than 90% of esophageal carcinoma cases [1].

Currently, surgery is the mainstay treatment for ESCC, but the overall treatment outcomes have not been satisfactory, with recurrence rates as high as 40–50% after radical surgery [2]. Locoregional recurrence is the most frequent recurrence pattern in ESCC even after definitive lymph node (LN) dissection [3, 4]. Several studies have shown that postoperative radiotherapy (PORT) can improve locoregional control in ESCC patients who undergo surgery [5–7]. However, these studies found no improved overall survival benefits among their total study cohorts and could therefore support no consensus for the use of PORT in ESCC [8]. However, a survival benefit was found in subgroup analysis, which indicated that further studies of PORT in this setting were warranted.

Defining reasonable target volume is very important in optimizing PORT, but no consensus on target volume for PORT in ESCC is yet available. We believe that identifying patterns of locoregional failure after surgery can help establish optimal PORT target volume, which prompted us to perform the present pooled analysis based on published data.

## Material and methods

We searched through PubMed for original investigations of patterns of failure after radical surgery in patients with ESCC that were published from May 1977 to March 2018. The PubMed database was chosen because it is the most widely used resource for medical literature and indexes only peer-reviewed biomedical literature.

The search strategy used the following key words in various combinations: “esophageal carcinoma”, “esophageal cancer”, “recurrence”, “LN”, “surgery”, and “resection”. The logic used for the search was “(((((esophageal cancer) OR esophageal carcinoma)) AND ((resection) OR surgery)) AND LN) AND recurrence)))))”. The inclusion criteria for the studies were (a) they described patterns of recurrence after radical surgery among patients with ESCC; (b) they should include patients with thoracic ESCC or predominantly thoracic ESCC, which would account for at least 90% of the study population; and (c) the studies were available as full texts, in English. We excluded studies in which one field lymphadenectomy or no lymphadenectomy was performed. If we found more than one article that used the same database, only the most suitable article was chosen for analysis. We also supplemented correlative articles by reading the references included in the reviews.

In our analysis, treatment failure was divided into local, regional, and distant failure. Local failure included esophagus, tumor bed, and anastomotic stoma. Regional failure included the regional LNs extending from peri-esophageal cervical to celiac LNs, according to the AJCC (7th edition) staging system; everything else was considered distant failure.

For regional failure, LN regions were first divided into cervical/supraclavicular, mediastinal and abdominal. For appropriate studies, we also performed analyses based on the mediastinal LN classification as upper, middle, and lower mediastinal LNs in parallel with the classification of their location at the esophagus, as described elsewhere [9]. The abdominal LN classification was divided into the upper abdominal and abdominal para-aortic LNs, as suggested by Doki et al. [10].

Whereas some previous studies enrolled only patients with recurrence, others offered continuous data from a consecutive series of patients with or without relapse. Therefore, we used two statistical variables: (a) recurrence rate, which was based on continuous patient data and represented the proportion of patients with recurrences at specific sites among all enrolled patients (with or without recurrence); and (b) the recurrence ratio, which represented the proportion of patients with recurrences at specific sites among all patients with recurrence. To determine the general recurrence rates of local, regional, and distant recurrences, we include studies that provided continuous patient data (including recurrent and non-recurrent patient data) and recurrence data for at least one of the LNs, local (tumor bed and anastomoses) and/or distal metastasis. Because most recurrences were in LNs, we explored the recurrence ratios of different LN regions using appropriate studies that reported total LN recurrence data and recurrence data for at least one of cervical/supraclavicular, upper mediastinal, middle mediastinal, lower mediastinal, abdominal paraaortic, and/or upper abdominal LNs. *P* < 0.05 was considered significant.

## Results

**Fig. 1:**
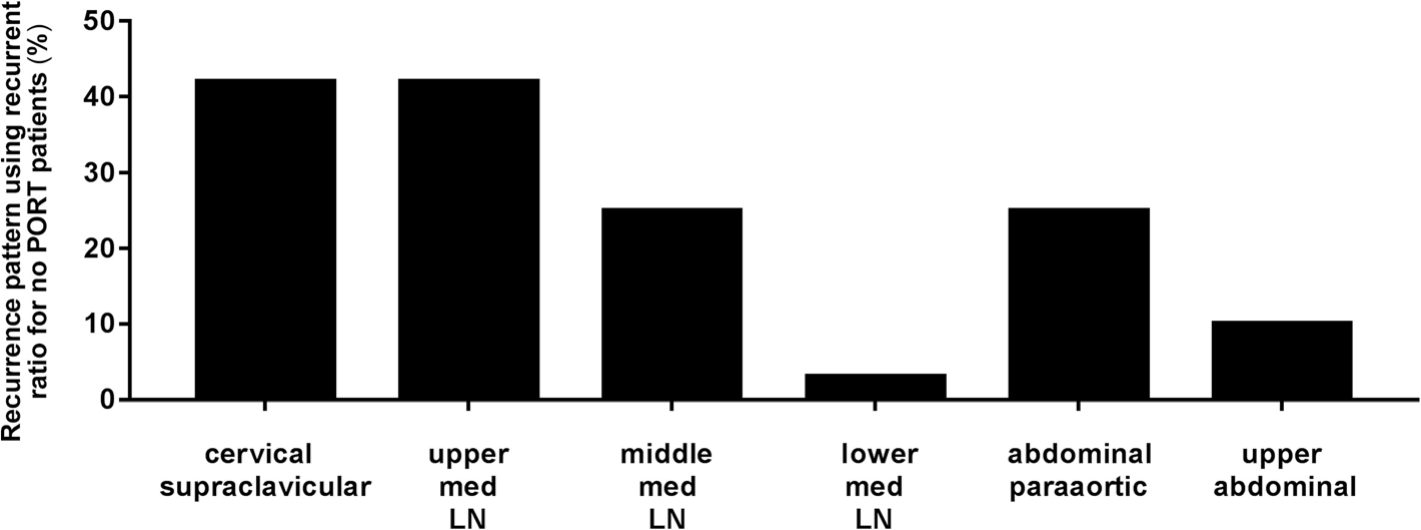
Flowchart of studies to final number of eligible studies

The initial search resulted in the identification of 1028 citations. The title and abstract of each retrieved publication were reviewed to confirm that the article reported on the incidence of recurrence patterns, including LN positivity in thoracic ESCC patients after radical surgery. If this approach was not informative, the full article was retrieved and reviewed in detail. We finally excluded 1012 studies and selected 14 studies (Fig. 1, Additional file 1: Table S1) [10–23], which included 2738 patients with thoracic ESCC who were treated with radical surgery, and of whom 1643 patients suffered recurrence or metastasis.

**Fig. 2:**
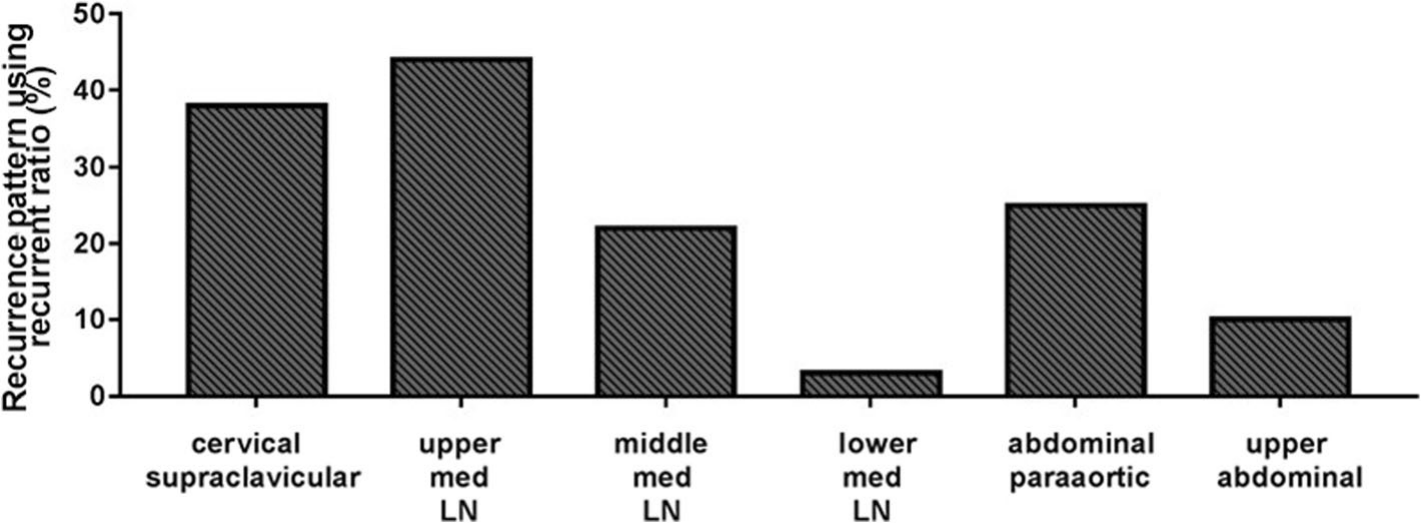
Recurrence pattern summary of recurrence ratio

Pooled analysis of the 10 selected studies showed that LN recurrence rates were regional: 22.16%, local: 1.88%, and distal: 13.18% (Table 1). Eleven studies were selected for calculating recurrence ratios for cervical/supraclavicular LNs [10–14, 17–22], 6 for upper mediastinal LNs [10–12, 14, 17, 22], 5 for middle and lower mediastinal LNs [10, 11, 14, 17, 22], and 2 for abdominal para-aortic and upper abdominal LNs (Table 2, Fig. 2) [10, 17].

**Table 1:** Recurrence pattern summary using recurrence rate

Study No.	Total sample size	LN Rec	Local	Distal Meta
2	90	18/90	2/90	20/90
3	414	160/414	13/414	49/414
4	501	121/501	13/501	72/501
6	112	37/112	1/112	7/112
8	70	15/70	*	*
9	196	51/196	1/196	44/196
10	174	34/174	1/174	29/174
11	685	74/685	19/685	47/685
12	208	29/208	8/208	32/208
rate%		22.16	1.88	13.18

*Not attainable from the literature

**Table 2:** Recurrence pattern summary using recurrence ratio

Study No.	LN Rec	Cervical supraclavicular LN	Upper Med LN	middle Med LN	lower Med LN	Abdominal paraaortic	Upper abdominal
1	126	55/126	93/126	50/126	2/126	*	*
2	18	3/18	3/18	*	*	*	*
3	160	61/160	*	*	*	*	*
4	121	34/121	30/121	9.5/121	9.5/121	30/121	8/121
5	79	35/79	18/79	21/79	2/79	*	*
8	15	10/15	6/15	3/15	0/15	4/15	5/15
9	51	8/51	*	*	*	*	*
10	34	10/34	*	*	*	*	*
11	74	50/74	*	*	*	*	*
12	29	3/29	*	*	*	*	*
13	106	37/106	56/106	14/106	0/106	*	*
ratio		317/841	206/465	97.5/447	11.5/447	34/136	13/136
%		37.69	44.30	21.81	2.57	25.0	9.56

*Not attainable from the literature

**Fig. 3:**
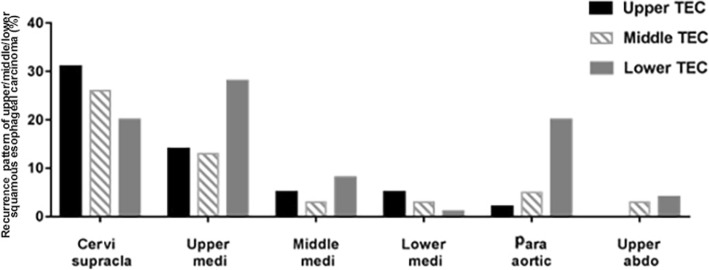
Recurrence pattern of upper/middle/lower squamous esophageal carcinoma using recurrence rate

Among studies that reported total recurrence data for patients and for the respective cervical, supraclavicular, upper mediastinal, middle mediastinal, lower mediastinal, abdominal paraaortic, and upper abdominal LN regions, two studies reported findings for upper thoracic ESCC [10, 13], three for middle thoracic ESCC, [10, 13, 23], and three for lower thoracic ESCC [10, 11, 22] (Table 3, Fig. 3).

**Table 3:** Recurrence pattern of upper/middle/lower squamous esophageal carcinoma using recurrence ratio

Study No.	Total Recurrence Size	Cervical supraclavicular LN	Upper Med LN	Middle Med LN	lower Med LN	Abdominal paraaortic	Upperabdominal
1	16	9/16	*	*	*	*	*
4	43	9/43	6/43	2/43	2/43	1/43	0/43
U(%)^		0.3051	0.1395	0.0465	0.0465	0.0233	0
1	92	40/92	*	*	*	*	*
4	141	20/141	19/141	4.5/141	4.5/141	7/141	4/141
14	338	96/338	*	88/338	*	*	*
M(%)^		0.2575	0.1348	0.0319	0.0319	0.0496	0.0284
1	18	6/18	*	*	*	*	*
4	109	5/109	5/109	3/109	3/109	22/109	4/109
13	108	37/108	56/108	14/108	0/108	*	*
L (%)^		0.2043	0.2811	0.0783	0.0138	0.2018	0.0367

*Not attainable from the literature. *U* upper, *M* middle, *L* lower

## Discussion

To our knowledge, this is the first pooled analysis of recurrence patterns after radical surgery among patients with thoracic ESCC. We found locoregional recurrence to be the most common recurrence pattern after radical surgery for thoracic ESCC at 24%, which was more than for distant metastases (13%), and is consistent with previous studies [19]. This finding indicates that PORT would useful in selected cases. Our study also showed that local sites (tumor beds and anastomoses) accounted for 1.88% of recurrences, compared with regional LNs at 22.16%, which implies that PORT should focus on regional LNs.

The esophagus lymphatic pathways consist of abundant lymphatics that form a dense submucosal plexus that transversally penetrates the esophageal wall and drains into adjacent LNs, and also has more longitudinal communication. This system is not segmental; therefore, LN metastases from thoracic ESCC tend to be widely dispersed [24–26]. With this in mind, a large T-shaped radiation field was first used, encompassing the tumor bed, bilateral cervical and supraclavicular areas, mediastinal LNs, site of anastomosis, and the left gastric LNs. However, such a large field can induce a relatively high rate of radiation toxicity [5]. Although several studies [7, 27–31] show the feasibility of using a reduced irradiation field in thoracic ESCC, no consensus on optimal target volume for PORT in thoracic ESCC has been reached.

Because surgical procedure would also influence the pattern of locoregional recurrence, we think that the locoregional recurrence pattern after surgery is more useful in guiding the PORT target designation directly than is the LN metastatic model. In this study, the cervical and supraclavicular areas and the upper mediastinal area had the highest recurrence rates compared with every other thoracic ESCC location. This might be partly because the incidences of LN metastasis was highest in all locations of the thoracic ESCC, and partly because of the difficulty of performing *en bloc* lymphadenectomies in the cervical and supraclavicular areas and in the upper mediastinal area, which are rich in nerves and large blood vessels. However, even the total incidence of LN metastases was high for the middle- and lower-thoracic ESCC, whereas incidences of recurrence in middle and lower mediastinum LNs were low. This might be partly because lymphadenectomy may be readily performed *en bloc* in the middle and lower mediastinum. In patients with lower thoracic ESCC, we found that the abdominal LN region was also an area of greater recurrence, which had recurrence rates similar to the cervical and supraclavicular region. However, when we performed further analysis, which divided the abdominal LNs into upper abdominal and abdominal para-aortic LNs, the abdominal para-aortic LN area was found to have a much higher recurrence rate than the upper abdominal LN area; this was also demonstrated in a phase III trial [5], in which the upper abdominal LN region was included in the PORT field, but recurrences in the upper abdominal LN were not reduced by PORT compared with surgery alone. This finding suggests that the upper abdominal LN region need not necessarily be encompassed in the PORT volume for thoracic ESCC.

Our study had some limitations. All of the studies included in this pooled analysis were retrospective; therefore, recurrence rates in different regions might not be accurate because of insufficient follow-up in some studies. To partly compensate for this fault for the LN recurrence pattern, we mainly used the recurrence ratio as the parameter that was analyzed only in recurrent patients, instead of using recurrence rates for all patients, which could avoid underestimation because of insufficient follow-up in some studies. We also could not explore predictive factors for locoregional recurrence based on the current information; thus, this study cannot provide information needed in selecting suitable patients for PORT. Although this subject is beyond the scope of the present study, additional studies, especially randomized clinical trials to explore predictive factors in selecting suitable patients for PORT, based on the normative irradiation target volume are warranted.

In our analysis, we included some studies in which some patients had received PORT, which might partly change the patterns of failure. However, because PORT has not been approved to be a worldwide standard of care for thoracic ESCC, these studies might have a variety of PORT target volume designs. Because we were not willing to omit high-risk LN recurrence areas even after PORT, we did not exclude the studies in which PORT was conducted only for some patients. Instead, we did further analyses based on studies with no PORT or PORT in not more than 10% of patients (Additional file 1: Tables S2–S4), and found the patterns of failure were not changed (Additional file 1: Figure S1).

In most of the included studies, the detailed information about postoperative chemotherapy were not provided. Although postoperative chemotherapy might reduce the local recurrence rate and metastasis rate for ESCC, we think postoperative chemotherapy will not significantly change spatial recurrence patterns, because chemotherapy is a systemic therapy that acts throughout the body. Furthermore, the systemic chemotherapy and local radiotherapy are not mutually exclusive; many patients need both after surgery.

## Conclusion

Taken together, we recommend that PORT volume include the cervical and supraclavicular LN areas and the upper mediastinal LN area for thoracic ESCC, as well as the abdominal para-aortic LNs for lower thoracic ESCC. The anastomoses and tumor beds should not be included in the PORT volume if they are not adjacent to the PORT-LN regions.

## Additional file

Additional file 1: **Table S1.** Eligible studies list. **Table S2.** Study list that containing patients number receiving postoperative radiation therapy. **Table S3.** Recurrence pattern summary using recurrence rate based on studies with no PORT or just doing the PORT in not more than 10% of patients. **Table S4.** Recurrence pattern summary using recurrence ratio based on the studies with no PORT or just doing the PORT in not more than 10% of patients. **Figure S1.** Recurrence pattern summary using recurrence ratio based on the studies with no PORT or just doing the PORT in not more than 10% of patients. (ZIP 143 kb)

## Abbreviations

EC: Esophageal carcinoma
ESCC: Esophageal squamous cell carcinoma
LNs: Lymph node
OS: Overall survival
PORT: Postoperative radiotherapy

## References

[1] Jemal A, Bray F, Center MM, Ferlay J, Ward E, Forman D (2011). Global cancer statistics. CA Cancer J Clin.

[2] Mirinezhad SK, Somi MH, Seyednezhad F, Jangjoo AG, Ghojazadeh M (2013). Survival in patients treated with definitive chemo-radiotherapy for nonmetastatic esophageal cancer in north- West Iran. Asian Pac J Cancer Prev.

[3] Giuli R, Gignoux M (1980). Treatment of carcinoma of the esophagus. Retrospective study of 2,400 patients. Ann Surg.

[4] Katayama A, Mafune K, Tanaka Y (2003). Autopsy findings in patients after curative esophagectomy for esophageal carcinoma. J Am Coll Surg.

[5] Xiao ZF, Yang ZY, Miao YJ (2005). Influence of number of metastatic lymph nodes on survival of curative resected thoracic esophageal cancer patients and value of radiotherapy: report of 549 cases. Int J Radiat Oncol Biol Phys.

[6] Chen JQ, Zhu J, Pan JJ (2010). Postoperative radiotherapy improved survival of poor prognostic squamous cell carcinoma esophagus. Ann Thorac Surg.

[7] Chen JQ, Pan JJ, Zheng XW (2012). Number and location of positive nodes, postoperative radiotherapy, and survival after esophagectomy with three-field lymph node dissection for thoracic esophageal squamous cell carcinoma. Int J Radiat Oncol Biol Phys.

[8] NCCN Clinical Practice Guidelines in Oncology: Esophageal and Esophagogastric JunctionCancers. Version 2.2018 -May 22, 2018. https://www.nccn.org/professionals

[9] Zhu Z, Yu W, Li H (2013). Nodal skip metastasis is not a predictor of survival in thoracic esophageal squamous cell carcinoma. Ann Surg Onco.

[10] Doki Y, Ishikawa O, Takachi K (2005). Association of the Primary Tumor Location with the site of tumor recurrence after curative resection of thoracic esophageal Carcinoma. World J Surg.

[11] Li C, Zhang F (2013). Characteristics of recurrence after radical esophagectomy with two-field lymph node dissection for thoracic esophageal cancer. Oncol Lett.

[12] Bhansali MS, Fujita H (1997). Pattern of recurrence after extended radical Esophagectomy with three-field lymph node dissection for squamous cell carcinoma in the thoracic esophagus. World J Surg.

[13] Liu Q, Cai X-W (2014). Patterns of failure after radical surgery among patients with thoracic esophageal squamous cell carcinoma: implications for the clinical target volume Design of Postoperative Radiotherapy. PLoS One.

[14] Guo X, Mao T (2015). Clinical study on postoperative recurrence in patients with pN1 esophageal squamous cell carcinoma. Thoracic Cancer.

[15] Guo X, Mao T (2014). Clinical study on postoperative recurrence in patients with pN0 esophageal squamous cell carcinoma. J Cardiothorac Surg.

[16] Chen X, Chen T-w (2014). Patterns of lymph node recurrence after radical surgery impacting on survival of patients with pT1-3N0M0 thoracic esophageal squamous cell carcinoma. J Korean Med Sci.

[17] Hiromasa Fujita MD, Teruo Kakegawa MD (1994). Lymph node metastasis and recurrence in patients with a carcinoma of the thoracic esophagus who underwent three-field dissection. World J Surg.

[18] Gang C, Wang Z (2006). Recurrence patterns of esophageal Cancer after Ivor-Lewis Esophagectomy—a report of 196 cases. Chinese Journal of Cancer.

[19] Nakagawa S, Kanda T (2004). Recurrence pattern of squamous cell carcinoma of the thoracic esophagus after extended radical Esophagectomy with three-field lymphadenectomy. J Am Coll Surg.

[20] Cai W-J, Xin P-L (2010). Pattern of relapse in surgical treated patients with thoracic esophageal squamous cell carcinoma and its possible impact on target delineation for postoperative radiotherapy. Radiother Oncol.

[21] Natsugoe S, Matsumoto M (2010). Clinical course and outcome after esophagectomy with three-field lymphadenectomy in esophageal cancer. Langenbeck's Arch Surg.

[22] Liu J, Cai X, Liu Q (2017). Characteristics of the local recurrence pattern after curative resection and values in target region delineation in postoperative radiotherapy for lower thoracic esophageal squamous cell cancer. Thoracic Cancer.

[23] Wang X, Luo Y, Li M (2016). Recurrence pattern of squamous cell carcinoma in the midthoracic esophagus: implications for the clinical target volume design of postoperative radiotherapy. OncoTargets and Therapy.

[24] Tachibana M, Kinugasa S, Shibakita M, Tonomoto Y, Hattori S (2006). Surgical treatment of superficial esophageal cancer. Langenbeck's Arch Surg.

[25] Chen J, Liu S, Pan J, Zheng X, Zhu K (2009). The pattern and prevalence of lymphatic spread in thoracic oesophageal squamous cell carcinoma. Eur J Cardiothorac Surg.

[26] Huang W, Li B, Gong H, Yu J, Sun H (2010). Pattern of lymph node metastases and its implication in radiotherapeutic clinical target volume in patients with thoracic esophageal squamous cell carcinoma: a report of 1077 cases. Radiother Oncol.

[27] Ténière P, Hay J-M, Fingerhut A (1991). Postoperation radiation therapy does not increase survival after curative resection for squamous cell carcinoma of the middle and lower esophagus as shown by a multicenter controlled trial. Surg Gynecol Obstet.

[28] Mei ZR, Xiang QC, Wu WJ (1997). Randomized controlled trial of prophylactic radiotherapy for postoperative esophageal carcinoma. Chin J Radiat Oncol.

[29] Fok M, Sham JS, Choy D (1993). Postoperative radiotherapy for carcinoma of the esophagus: a prospective, randomized controlled study. Surgery.

[30] Bédard EL, Inculet RI, Malthaner RA (2001). The role of surgery and postoperative chemoradiation therapy in patients with lymph node positive esophageal carcinoma. Cancer.

[31] Nishimura Y, Ono K, Imamura M (1989). Postoperative radiation therapy for esophageal cancer. Radiat Med.

