# Supplementary material for: Recommendation for the definition of postoperative radiotherapy target volume based on a pooled analysis of patterns of failure after radical surgery among patients with thoracic esophageal squamous cell carcinoma
Source: Radiat Oncol. 2018 Dec 27;13:255. doi: 10.1186/s13014-018-1199-3 (PMC6307221; doi:10.1186/s13014-018-1199-3)
Supplement: Supplementary file 1 — Table S1. Eligible studies list. Table S2. Study list that containing patients number receiving postoperative radiation therapy. Table S3. Recurrence pattern summary using recurrence rate based on studies with no PORT or just doing the PORT in not more than 10% of patients. Table S4. Recurrence pattern summary using recurrence ratio based on the studies with no PORT or just doing the PORT in not more than 10% of patients. Figure S1. Recurrence pattern summary using recurrence ratio based on the studies with no PORT or just doing the PORT in not more than 10% of patients. (ZIP 143 kb) [file 13014_2018_1199_MOESM1_ESM.zip › Table supplement.docx]

**Figure Legend for supplement figure**

**Figure S1**

Recurrence pattern summary using recurrence ratio based on the studies with no PORT or just doing the PORT in not more than 10% of patients

**Table supplement**

Table S1 Eligible studies list.

| **No.** | **study** | **Year** |  | **sample size** | **Total recurrence** |
| --- | --- | --- | --- | --- | --- |
| 1 | Chenglin Li[11] | 2013 | ONCOLOGY LETTERS | * | 134 |
| 2 | Mehul S[12] | 1997 | World J. Surg. | 90 | 40 |
| 3 | Qi Liu[13] | 2014 | https://doi.org/10.1371/journal.pone.0097225 | 414 | 207 |
| 4 | Yuichiro Doki[10] | 2005 | World J. Surg. | 501 | 180 |
| 5 | Xufeng Guo[14] | 2014 | Thoracic Cancer | * | 97 |
| 6 | Xufeng Guo[15] | 2014 | Journal of Cardiothoracic Surgery | 112 | 45 |
| 7 | Xiaoli Chen[16] | 2014 | J Korean Med Sci | 180 | * |
| 8 | Hiromasa Fujita[17] | 1994 | World J. Surg. | 70 | 24 |
| 9 | Chen Gang[18] | 2006 | Chinese Journal of Cancer, | 196 | 96 |
| 10 | Satoru Nakagawa[19] | 2004 | J Am Coll Surg | 174 | 74 |
| 11 | Wen-Jie Cai[20] | 2010 | Radiotherapy and Oncology | 685 | 140 |
| 12 | Shoji Natsugoe[21] | 2010 | Langenbecks Arch Surg | 208 | 104 |
| 13 | Jun Liu[22] | 2017 | Thoracic Cancer | * | 108 |
| 14 | Xiaoli Wang[23] | 2016 | OncoTargets and Therapy | * | 338 |

Table S2 Study list that containing patients number receiving postoperative radiation therapy

| Study  number | Author  [Reference number] | Total size number | Post operative radiation number |
| --- | --- | --- | --- |
| 1 | Chenglin Li[11] | 134 | 0 |
| 2 | Mehul S[12] | 90 | 6 |
| 3 | Qi Liu[13] | 414 | 0 |
| 4 | Yuichiro Doki[10] | 501 | 3 |
| 5 | Xufeng Guo[14] | 95 | 0 |
| 6 | Xufeng Guo[15] | 112 | 0 |
| 7 | Xiaoli Chen[16] | 180 | 18 |
| 8 | Hiromasa Fujita[17] | 70 | 6 |
| 9 | Chen Gang[18] | 196 | 60 |
| 10 | Satoru Nakagawa[19] | 174 | 42 |
| 11 | Wen-Jie Cai[20] | 685 | 0 |
| 12 | Shoji Natsugoe[21] | 199 | Not mentioned |
| 13 | Jun Liu[22] | 108 | Not mentioned |
| 14 | Xiaoli Wang[23] | 338 | 0 |

Table S3 Recurrence pattern summary using recurrence rate based on studies with no PORT or just doing the PORT in not more than 10% of patients

| Study  No. | Total sample size | LN  Rec | Local | Distal  Meta |
| --- | --- | --- | --- | --- |
| 2 | 90 | 18/90 | 2/90 | 20/90 |
| 3 | 414 | 160/414 | 13/414 | 49/414 |
| 4 | 501 | 121/501 | 13/501 | 72/501 |
| 6 | 112 | 37/112 | 1/112 | 7/112 |
| 8 | 70 | 15/70 | * | * |
| 11 | 685 | 74/685 | 19/685 | 47/685 |
| rate |  | 425/1872 | 48/1802 | 195/1802 |
| rate% |  | 22.70 | 2.66 | 10.82 |

*Not attainable from the literature

Table S4 Recurrence pattern summary using recurrence ratio based on the studies with no PORT or just doing the PORT in not more than 10% of patients

| Study  No. | LN  Rec | Cervical supraclavicular  LN | Upper  Med  LN | middle  Med  LN | lower  Med  LN | Abdominal paraaortic | Upper abdominal |
| --- | --- | --- | --- | --- | --- | --- | --- |
| 1 | 126 | 55/126 | 93/126 | 50/126 | 2/126 | * | * |
| 2 | 18 | 3/18 | 3/18 | * | * | * | * |
| 3 | 160 | 61/160 | * | * | * | * | * |
| 4 | 121 | 34/121 | 30/121 | 9.5/121 | 9.5/121 | 30/121 | 8/121 |
| 5 | 79 | 35/79 | 18/79 | 21/79 | 2/79 | * | * |
| 8 | 15 | 10/15 | 6/15 | 3/15 | 0/15 | 4/15 | 5/15 |
| 11 | 74 | 50/74 | * | * | * | * | * |
| ratio |  | 248/593 | 150/359 | 83.5/341 | 11.5/341 | 34/136 | 13/136 |
| % |  | 41.82 | 41.80 | 24.51 | 3.37 | 25.00 | 9.56 |

*Not attainable from the literature
