# Supplementary figures and images for: Recommendation for the definition of postoperative radiotherapy target volume based on a pooled analysis of patterns of failure after radical surgery among patients with thoracic esophageal squamous cell carcinoma
Source: Radiat Oncol. 2018 Dec 27;13:255. doi: 10.1186/s13014-018-1199-3 (PMC6307221; doi:10.1186/s13014-018-1199-3)

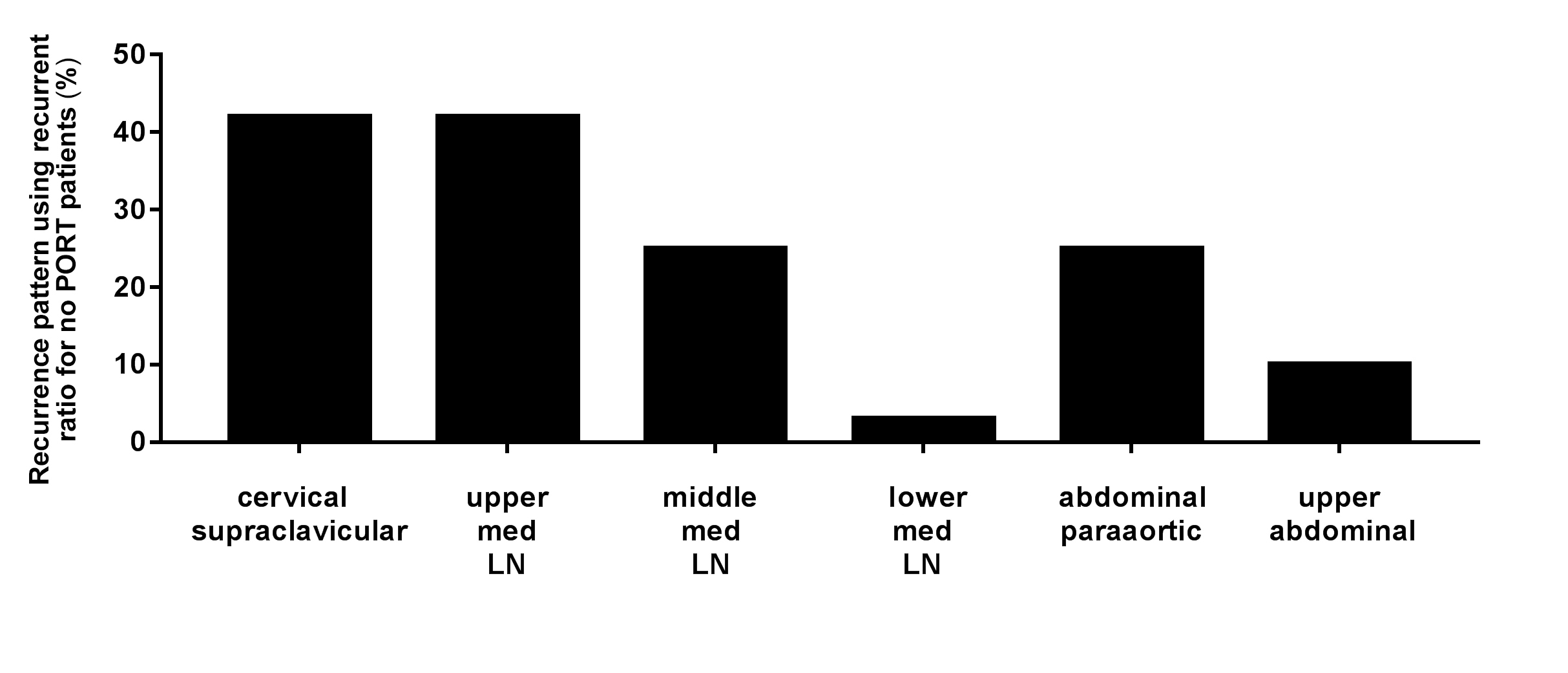

Supplement: Supplementary file 1 — Table S1. Eligible studies list. Table S2. Study list that containing patients number receiving postoperative radiation therapy. Table S3. Recurrence pattern summary using recurrence rate based on studies with no PORT or just doing the PORT in not more than 10% of patients. Table S4. Recurrence pattern summary using recurrence ratio based on the studies with no PORT or just doing the PORT in not more than 10% of patients. Figure S1. Recurrence pattern summary using recurrence ratio based on the studies with no PORT or just doing the PORT in not more than 10% of patients. (ZIP 143 kb) [file 13014_2018_1199_MOESM1_ESM.zip › Fig supplement 1 NEW.jpg]
